# Effects of Eight Weeks of Selected Virtual-Assisted Montessori-Based Games on Motor Proficiency and Perceived Self-Control in Children with Spastic Hemiplegia during the Coronavirus Outbreak

**DOI:** 10.1155/2022/5792094

**Published:** 2022-12-06

**Authors:** Mahsa Khaledi, Ali Heirani, Ayoob Sabaghi

**Affiliations:** Department of Sport Sciences, Razi University, Kermanshah, Iran

## Abstract

This study is aimed at examining the effect of eight weeks of selected virtual-assisted games based on Montessori pedagogical principal on the motor proficiency and perceived self-control in the children with spastic hemiplegia during the coronavirus outbreak. In this quasi-experimental study, the children (6.42*y* ± 1.12*y*) with hemiplegia were randomly selected and assigned to either the experimental group or the control group. In the pretest, motor skills and self-controlling were evaluated using 36-item Lincoln-Oseretsky Motor Development Scale and Children's Perceived Self-Control Scale (CPSC) (ss, 1982), respectively. The experimental group then engaged in three 45-minute sessions of virtual game play over the course of eight weeks. 24 hours following the last practice session, the posttest was given on the same day as the pretest. After ensuring the normal distribution of collected data with Shapiro-Wilk test, the data were analyzed using Analysis of Covariance test (ANCOVA). Results showed that the experimental group compared to the control group was better in the motor proficiency and perceived self-control after performing selected virtual-assisted Montessori games can significantly improve motor proficiency and perceived self-control (*P* < 0.05). This pattern of data revealed that the virtual-assisted intervention based on Montessori pedagogical principles may increase motor proficiency and self-control in children with hemiplegia, particularly when confronted with the limitations imposed on by the coronavirus epidemic.

## 1. Introduction

The coronavirus (COVID-19) causes the respiratory tract infection. In 2020, World Health Organization (WHO) introduced this disease as a major threat to physical and mental health because the lifestyle was seriously changed during this epidemic [1, 2]. School closures and home-quarantine of children have adversely affected the children's physical health in terms of reduced physical activity and the lack of adequate space for physical activity [3]. Ghosh et al. [4] found that at the times of crisis, especially epidemics, children are more likely to encounter serious problems. The coronavirus epidemic causes greater issues for unhealthy children than for the healthy children [5]. For instance, during the coronavirus outbreak, children with cerebral palsy were among those who had a multitude of issues. The term “CP” refers to a group of persistent, nonprogressive, and developmental motor deficits in the posture that are connected to behavioral, cognitive, and communicative problems that may appear both before and shortly after birth [6]. These children have many difficulties in terms of their rehabilitation and training sessions. These children often forced to stay at home for long periods of time due to compulsory isolation and school closures, which resulted in limited contact with their partners and reduced physical activity. Thus, more behavioral and emotional problems appeared among them [7, 8]. The level of functional independence in daily activities of children with CP is limited by these disorders [9]. They disrupt performance and motor skills, which affects one's ability in most activities, such as daily life activities, self-care, mobility, and communications. Therefore, their engagement in motor skills is reduced [10]. Common treatments for CP include occupational therapy, physiotherapy, medication therapy, and orthopedic surgeries [11]. Spastic hemiplegic is a type of CP, which engages one side of the body. According to researchers, if enough opportunities for exercise and physical activity can be provided, motor functions in the children with spastic hemiplegic cerebral palsy (SHCP) may improve [12]. Vernadakis et al. [13] evaluated the effect of exergame-based intervention on early elementary school children's motor skills and showed that this intervention had significant effects. Peres et al. [14] examined the effect of play therapy techniques on the motor rehabilitation of CP children in a different research. Their study's findings suggested that these children's motor abilities had improved. The children with this disorder struggle with severe self-control issues. Self-control is the ability to regulate one's thoughts, feelings, and actions. Self-control is often defined as the ability to manage impulsive behavior by suppressing short-term, urgent desires [15]. Self-control is often seen as a very consistent personality attribute that is connected to a wide range of activities [16]. People with strong self-control are more able to contain their thoughts, manage their emotions, and limit their motives than individuals with poor self-control [17]. They experience greater levels of accomplishment, stronger interpersonal relations, and better mental health [18]. High self-control has become an important term in many study fields since it is roughly connected with many types of behavior, which contribute to a successful and healthy life [17]. Research studies showed that games and physical activities result in the biochemical balance of brain.

Montessori games are a pedagogical approach, which was developed and introduced by an Italian physician named Montessori. She created a variety of original tools and educational strategies for educating children utilizing their own senses. In general, the Montessori method of instruction differs significantly from other game-based educational approaches. The Montessori educational philosophy seeks to broaden the scope of the autonomous learning environment and enhance reading, math, executive, and social comprehension skills in students [19]. However, up to now, very few research studies were done on the effects of Montessori games on motor proficiency and self-control. Kaya and Yildiz [20] evaluated the beneficial effects of a course of selected Montessori games on the motor proficiency of trainable mentally disabled children. The results of their study indicated the positive effects of these games on motor proficiency. Foreman found a significant improvement in children's self-control when she evaluated the effect of the group games with a Montessori foundation on self-controlled conduct [21]. The child actively participates in all daily activities according to the Montessori educational technique [22]. The Montessori exercises are based on kid-friendly physical and mental games, and they are designed to encourage the child's active involvement in all daily activities [23]. Therefore, it is anticipated that these activities would improve children with HCP's motor skills and self-control. Thus, the effects of this type of virtual-assisted intervention on different developmental aspects of children were not evaluated, but other research studies on the effects of virtual reality-based intervention on functional aspects of children indicated the beneficial effects of this method [24, 25]. Therefore, the selected virtual-assisted Montessori games are expected to have beneficial effects on these children. Thus, this study is aimed at determining how 8 weeks of playing a few virtual-assisted Montessori games affected the children with spastic hemiplegia's motor skills and self-control during the coronavirus epidemic.

## 2. Methods

### 2.1. Methods and Statistical Population

In this semiexperimental study, the research population consisted of all 5-8-year-old (6.42 ± 1.12) children with spastic hemiplegia in Kermanshah. 24 children were randomly selected from rehabilitation centers and based on the pretest results assigned into two same groups as experimental group and control group. The selected sample size was selected based on almost similar studies that were conducted virtually [26, 27].

Written informed consent to participate in the study was obtained from the children's parents, and the measurement sessions were held in conformity with health protocols. This research was conducted based on Code of Ethics approved by the Ethics Committee of Razi University of Kermanshah, Iran (IR.RAZI.REC. 1400.079).

Inclusion criteria were as follows: the children with spastic hemiplegia, not undergoing surgery, not experiencing this training program or similar physical activities, without visual problems or with modified eyesight (eyeglasses), not suffering from other diseases, such as epilepsy and intellectual disability, having no pain in the nonaffected organ.

Participation in any activity not indicated by the researcher and missing two consecutive or three alternative training sessions were also forbidden. Developing a condition that makes it physically unable to do the activities while the intervention technique is being used. Two participants from the control group were excluded from the research plan due to participating in the activities outside of intended programs and another participant from the experimental group in terms of relocation and lack of access to the Internet and receiving exercise programs. The key inclusion criterion was the absence of any participant activity other than what was indicated by the researcher; therefore, the parents were asked to report any activity outside of the children's program. As a consequence, the internal validity of the research is supported. The participants in the control group, in addition to participating in the pre- and posttest, were informed about the benefits of exercise and physical activity by the researchers in the form of a video call and at the same time as the experimental group.

### 2.2. Instruments

#### 2.2.1. Motor Proficiency

Lincoln-Oseretsky Test of Motor Proficiency was used to measure motor proficiency in children. This scale was designed to assess motor proficiency in 5-14-year-old children and contains 36 items. Each item was scored based on a three-point rating scale from 0 to 2, and the final total score would be 159. The provided scores were plotted, and the resulting standard table displays a person's standing in terms of being normal or atypical. By examining the correlations between the test's subscales and overall score, it was possible to determine that this test's Cronbach's alpha reliability was 0.73 and its validity was 0.82. This test is a developmental scale [28].

#### 2.2.2. Self-Control Measurement

The interpersonal self-control (ISC), personal self-control (PSC), and self-evaluation (SE) subscales of the Children's Perceived Self Control Scale (CPSC), which is based on the original Humphrey [29] scale, each include a total of 11 items. Children are presented with the scale under the title of “Usually That's Me” and asked to respond either “Usually Yes” or “Usually No” according to how well the questions describe them. Reliability estimations produced a correlation of 71 for the overall scale, with subscale correlations of 63 for ISC, 63 for PSC, and 56 for SE. Naturalistic observations have also shown the criterion-related validity of these reliability estimates. Additionally, this questionnaire has been utilized in several studies to assess self-control [30–32].

### 2.3. Procedure

**Figure 1:**
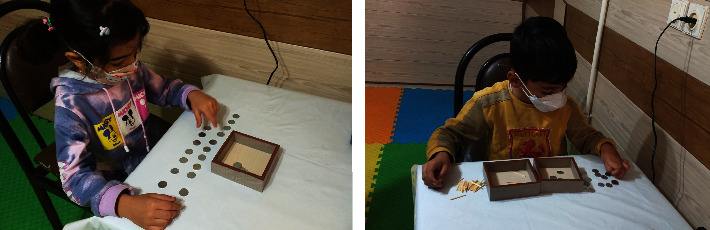
An example of the tests performed in the present study.

After the sampling process, the participants were randomly assigned either to the control group or the experimental group in pretest both groups conducted the 36-item Lincoln-Oseretsky Motor Development Scale and Children's Perceived Self-Control Scale (CPSC), respectively. Then, the experimental group performed Montessori education program (Table 1). For eight weeks, the participants in the experimental group exercised three times per week while playing the selected virtual Montessori motor exercises. Each home workout session began with a five-minute warm-up, followed by the primary exercises listed in Table 1. The exercises were designed from simple to advanced level during the sessions. The participants in the experimental group were communicated using group video calls in virtual social networks at a specific time, and cooling down was performed for 5 minutes in the end. A posttest was then administered the same as the pretest after the interventions (Figure 1). The materials required for Montessori games were supplied to the participants at the beginning of each session, and they were instructed on how to use it and complete activities. Researchers and children spoke via video calls on the virtual network WhatsApp. The participants appeared in front of the camera at the appointed time and performed the exercises in front of the researchers with full clarity for a certain period of time, any errors and deficiencies in the participants' exercises were observed by the researchers, and feedback was given to the participants.

### 2.4. Statistical Analysis

In the descriptive statistics, mean and standard error were used. After confirming the data distribution, analysis of covariance (ANCOVA) was used for data analysis. The data were analyzed using SPSS.23 software at the significance level of *P* < 0.05.

## 3. Results

The descriptive results of variables related to the motor proficiency and perceived self-control tests are presented in Table 2 for the control and experimental groups. ANCOVA test was used to assess the impact of chosen Montessori games on the dependent variables of the study. The effect of certain Montessori game treatments on the study's dependent variables was examined using the ANCOVA test. This test's test assumptions were examined before it was run. To determine if the sample distribution was normal, the Shapiro-Wilk test was calculated. Levin test was used to evaluate the homogeneity of variance of the research variables. Preliminary analysis of the data with abovementioned tests confirmed the normality of data distribution and equality of variances (*P* > 0.05).

ANCOVA results are shown in Table 3. As shown in table, the selected virtual Montessori games program significantly affected motor proficiency and self-control in the children with spastic hemiplegia (*P* < 0/05).

## 4. Discussion

In critical and emergency situations, such as the outbreak of COVID-19, the effects of the disease not only lead to reduced physical activity but also cause mental health problems. This can adversely affect self-control. Maynard et al. stated that people, especially children, faced many problems during the outbreak of COVID-19, which can lead to stress, frustration, and decreased levels of self-control in children [33]. During the coronavirus epidemic, this research was designed to evaluate the effects of a selected virtual aided intervention based on Montessori games on motor proficiency and self-control in kids with spastic hemiplegia. Numerous issues arose during the prevalence of the corona virus, especially in children with HCP. As compared to the control group, acquired findings showed a substantial increase in motor competence and self-control in the experimental group during the coronavirus prevalence. Research similar to the present research that dealt with the intervention method with virtual assistance on motor competence and self-control was not observed, but was consistent with the research, which was done based on virtual reality [26, 27]. For example, Zhanbing and Jinlang [24] evaluated the effect of virtual reality games on gross motor skills in the children with CP. The findings of their review suggested that virtual games had a good impact on these abilities. Silvia et al. [25] evaluated the impact of a virtual reality-based intervention on hemiplegic children's motor function and balance. The results of their study showed the desirable effects of virtual interventions on children with hemiplegia. The advantage of methods based on Virtual Assisted Intervention can be the coach or teacher can give the learner the necessary feedback if he performs the movement incorrectly and correct the movement of the learner, and it seems that it can have more effects compared to the reality of the virtual space.

The findings of the current study, which used virtual help to carry out its investigation, were consistent with other investigations that looked at how the Montessori method's games and physical activities affected children's functional qualities in real space. According to Kayili's [34] analysis of the effects of the Montessori Method on preschoolers' ready for primary education, the Montessori program significantly improves these kids' readiness for primary school and is more effective than the existing preschool program. In another study, Kaya and Yildiz [20] evaluated the effect of Montessori programs on the motor proficiency of trainable mentally disabled children. The results of their study indicated the beneficial effects of the selected Montessori games on the motor proficiency of these children. Lillard [35] states that practical Montessori programs are efficient because they make tasks easy for children, provide immediate feedback, and encourage learning. Therefore, it can be said that the Montessori program significantly affects the learning process. Moreover, Bhatia et al. [36] designed a study to evaluate the effect of Montessori life activities on motor proficiency. The results of their study indicated the improvement of motor proficiency in the experimental group.

Moreover, we observed an improved level of self-control in the experimental group of children with hemiplegia. Baggerly and Parker [37] stated that group play therapy has beneficial effects on self-control, acceptance of self and others, improvement of social skills, and reduction of depression.

Gamma aminobutyric acid (GABA) is a nerve stimulator, which is crucial to control the mental and mental impulses. It is better controlled by games and physical activity, which helps relax the brain and somewhat improve self-control [38]. An improvement in self-control can be attributed to the improvement of motor proficiency besides neural mechanisms [39]. It was observed that children with higher motor proficiency are more likely to perform daily activities successfully and, thus, develop their self-control [40]. Moreover, in game therapy, children can show their concerns in a safe psychological environment and become aware of their abilities, limitations, rules, and realities; practice new skills; and discover creative solutions to their challenges to cope with difficult situations [41].

According to Goodman and Stackler [42], Montessori education reflects a flexible and compatible model. Montessori pedagogy method is characterized by unstructured games and activities in a suitable setting. This educational environment may provide corresponding spaces to meet the needs of children of different ages [43]. The benefit of this educational approach was even virtually noticed in the children with hemiplegia, and the Montessori activity is a manifestation of various well-known features to improve learning and development [44].

## 5. Conclusion

In general, and based on the results of present study, this pedagogical method can affect the improvement of motor proficiency and self-control in the children with hemiplegia. It is suggested that educators who deal with children with hemiplegia include the selected Montessori-based games used in their curriculum, either virtually or in-person where in-person attendance is possible.

## Figures and Tables

**Table 1:** The games used in research based on Montessori education method.

Sessions	Main interventions		
1st session	Wrist flexion and extension	Paper crumpling	Rolling the ball with the foot
2nd session	Squeezing the sponge ball	Putting together related shapes and with the same color	Picking up toys from the floor without falling
3rd session	Crumpling the paper and smoothing it	Pickup*-*object and drop*-*object in the box	Throwing the ball with hands and feet towards the target
4th session	Squeezing jelly balls with fingers	Folding a bath towel and clothes on the table	Playing hide and seek
5th session	Connecting the dots on the paper	Scissoring the papers based on the pattern depicted	Finding the hidden objects
6th session	Putting colored sticks in cans of the same color	Drawing different shapes such as circles, etc.	Throwing the ball down into the basket by hand
7th session	Scissoring the papers based on the pattern depicted	Rolling the ball with the foot	Playing hide and seek
8th session	Putting together related shapes and with the same color	Pickup*-*object and drop*-*object in the box	Finding the hidden objects

**Table 2:** Mean and standard error of groups in research tests.

Variable	Experimental group		Control group	
	Pretest	Posttest	Pretest	Posttest
Motor proficiency	44.63 ± 4.84	51.45 ± 4.16	44.30 ± 3.06	44.60 ± 2.92
Self-control	6.27 ± 0.54	7.18 ± 0.58	5.40 ± 0.45	5.60 ± 0.42

**Table 3:** ANCOVA results to compare the performance of two groups on the research tests.

Variable	df	*F*	Sig	Partial eta	Statistical power
Motor proficiency	1	14.56	0.001	0.461	0.949
Self-control	1	5.90	0.026	0.247	0.633

## Data Availability

The datasets used and/or analyzed during the current study are available from the corresponding author on reasonable request.
